# Excitation-Contraction Coupling in Zebrafish Ventricular Myocardium Is Regulated by Trans-Sarcolemmal Ca^2+^ Influx and Sarcoplasmic Reticulum Ca^2+^ Release

**DOI:** 10.1371/journal.pone.0125654

**Published:** 2015-05-04

**Authors:** Moritz Haustein, Tobias Hannes, Jan Trieschmann, Rabea Verhaegh, Annette Köster, Jürgen Hescheler, Konrad Brockmeier, Roland Adelmann, Markus Khalil

**Affiliations:** 1 Department of Paediatric Cardiology, Cologne Heart Centre, Medical Faculty, University of Cologne, Cologne, North Rhine-Westphalia, Germany; 2 Institute for Neurophysiology, Medical Faculty, University of Cologne, Cologne, North Rhine-Westphalia, Germany; 3 Institute of Physiological Chemistry, University Hospital Essen, Essen, North Rhine-Westphalia, Germany; 4 Department of Paediatric Cardiology, University Hospital of Giessen and Marburg, University of Giessen and Marburg, Giessen, Hessen, Germany; Brigham & Women's Hospital - Harvard Medical School, UNITED STATES

## Abstract

Zebrafish (*Danio rerio*) have become a popular model in cardiovascular research mainly due to identification of a large number of mutants with structural defects. In recent years, cardiomyopathies and other diseases influencing contractility of the heart have been studied in zebrafish mutants. However, little is known about the regulation of contractility of the zebrafish heart on a tissue level. The aim of the present study was to elucidate the role of trans-sarcolemmal Ca^2+^-flux and sarcoplasmic reticulum Ca^2+^-release in zebrafish myocardium. Using isometric force measurements of fresh heart slices, we characterised the effects of changes of the extracellular Ca^2+^-concentration, trans-sarcolemmal Ca^2+^-flux via L-type Ca^2+^-channels and Na^+^-Ca^2+^-exchanger, and Ca^2+^-release from the sarcoplasmic reticulum as well as beating frequency and β-adrenergic stimulation on contractility of adult zebrafish myocardium. We found an overall negative force-frequency relationship (FFR). Inhibition of L-type Ca^2+^-channels by verapamil (1 μM) decreased force of contraction to 22±7% compared to baseline (n=4, p<0.05). Ni^2+^ was the only substance to prolong relaxation (5 mM, time after peak to 50% relaxation: 73±3 ms vs. 101±8 ms, n=5, p<0.05). Surprisingly though, inhibition of the sarcoplasmic Ca^2+^-release decreased force development to 54±3% in ventricular (n=13, p<0.05) and to 52±8% in atrial myocardium (n=5, p<0.05) suggesting a substantial role of SR Ca^2+^-release in force generation. In line with this finding, we observed significant post pause potentiation after pauses of 5 s (169±7% force compared to baseline, n=8, p<0.05) and 10 s (198±9% force compared to baseline, n=5, p<0.05) and mildly positive lusitropy after β-adrenergic stimulation. In conclusion, force development in adult zebrafish ventricular myocardium requires not only trans-sarcolemmal Ca^2+^-flux, but also intact sarcoplasmic reticulum Ca^2+^-cycling. In contrast to mammals, FFR is strongly negative in the zebrafish heart. These aspects need to be considered when using zebrafish to model human diseases of myocardial contractility.

## Introduction

The popularity of the Zebrafish (*Danio rerio*) as a model organism in cardiovascular research is a result of features such as a high degree of genetic homology with mammals and especially humans, ease of handling and reproduction, and straightforward techniques for genetic manipulation like gene-specific antisense knockdown using morpholino oligonucleotides [1]. A major experimental advantage is that, especially in early stages of development, the transparency of zebrafish embryos permits *in vivo* imaging of cardiovascular structures [1].

These experimental advantages are opposed by profound anatomical differences between the zebrafish and the human heart. A key distinction is that the zebrafish heart does not undergo septation. Thus, it remains two-chambered and is composed of an inflow tract, *sinus venosus*, a single atrium, and a single ventricle which is separated from the atrium by endocardial cushions. In addition, ventricular myocardium of the zebrafish consists of an outer compact layer and a trabeculated inner layer [2] while the healthy human left ventricle is characterised by the compactness of the myocardial wall. On the cellular level, zebrafish ventricular cardiomyocytes lack a t-tubular system [3–4] which is an essential component of excitation-contraction (EC) coupling in the mammalian heart.

One might argue that these prominent structural differences must be accompanied by functional differences of the same order. Surprisingly, with regard to electrophysiological function, the two-chambered zebrafish heart shares many properties with the human heart. Zebrafish possess a cardiac conduction system similar to the human heart and the heart rate is controlled by a group of pacemaking cells in the sinoatrial region [5]. Heart rate [6] as well as shape of the ventricular action potential [3] are similar to the human correlate. This is in stark contrast with other model systems such as mice whose heart rates and repolarisation characteristics fundamentally differ from the human heart. Thus, zebrafish are an interesting and complementary model to study electrophysiological disorders such as the long QT syndrome [7].

Moreover, zebrafish have amply been used to study disorders of contractility such as dilated cardiomyopathy. Mutations in components of the contractile apparatus like titin [8], tropomyosin [9], troponin T type 2 [10], myosin light-chain-2 [11] were shown to lead to ventricular dilation and reduced contractile performance.

Whether zebrafish is however a truly attractive model to study contractility and contractile dysfunction depends on how close zebrafish cardiomyocytes mimic EC coupling of the mammalian heart. In mammals [12], electrical excitation leads to influx of extracellular calcium through the L-type Ca^2+^ channel (LTCC). This leads to a local rise of the calcium concentration which activates the ryanodine receptor (RyR) in the membrane of the sarcoplasmic reticulum (SR) to release a greater amount of Ca^2+^. This process of Ca^2+^-induced Ca^2+^-release (CICR) requires however a direct interface between the cell and the SR membrane which is enabled in mammalian cardiomyocytes by invaginations of the cell membrane, so called t-tubules. Ca^2+^ is mainly removed from the cytosol by reuptake into the SR as well as trans-sarcolemmal Ca^2+^-efflux through the Na^+^-Ca^2+^-exchanger (NCX) and a sarcolemmal Ca^2+^-ATPase.

Fundamental differences in Ca^2+^-cycling seem to exist between mammalian and zebrafish cardiomyocytes. T-tubules are absent in zebrafish cardiomyocytes [3–4] and accordingly trans-sarcolemmal Ca^2+^-influx is reported to be the main contributor to the Ca^2+^-transient while CICR from the SR seems to play a lesser role [4, 13]. It has however not been clarified to what extent these differences impact on the regulation of force development in the zebrafish heart. Therefore, in the present study, we characterised the contractile behaviour of the zebrafish ventricle using a heart slice model established in our group [14]. Specifically, we investigated the effects of changing contraction frequency, of modulating extracellular calcium and calcium influx, of inhibiting SR Ca^2+^-release, of inhibiting NCX, and of β-adrenergic stimulation on isometric contractions.

## Materials and Methods

### Animals

Zebrafish (Cologne wild-type strain) stocks were maintained at 27.2°C at 12-h light/12-h dark cycle. Fish were fed with flake food, brine shrimps and lobster eggs. Adult wild-type zebrafish of both sexes were used at the age of 3–4 months for all experiments. All animal procedures were in accordance to the Guidelines of the animal welfare committee of the University of Cologne and were approved by the North Rhine-Westphalia State Agency for Nature, Environment and Consumer Protection (LANUV, approval no. 84–02.05.20.13.020). Schedule 1 killing was performed under general anaesthesia, and all efforts were made to minimise suffering.

### Preparation of ventricular tissue slices

**Fig 1:**
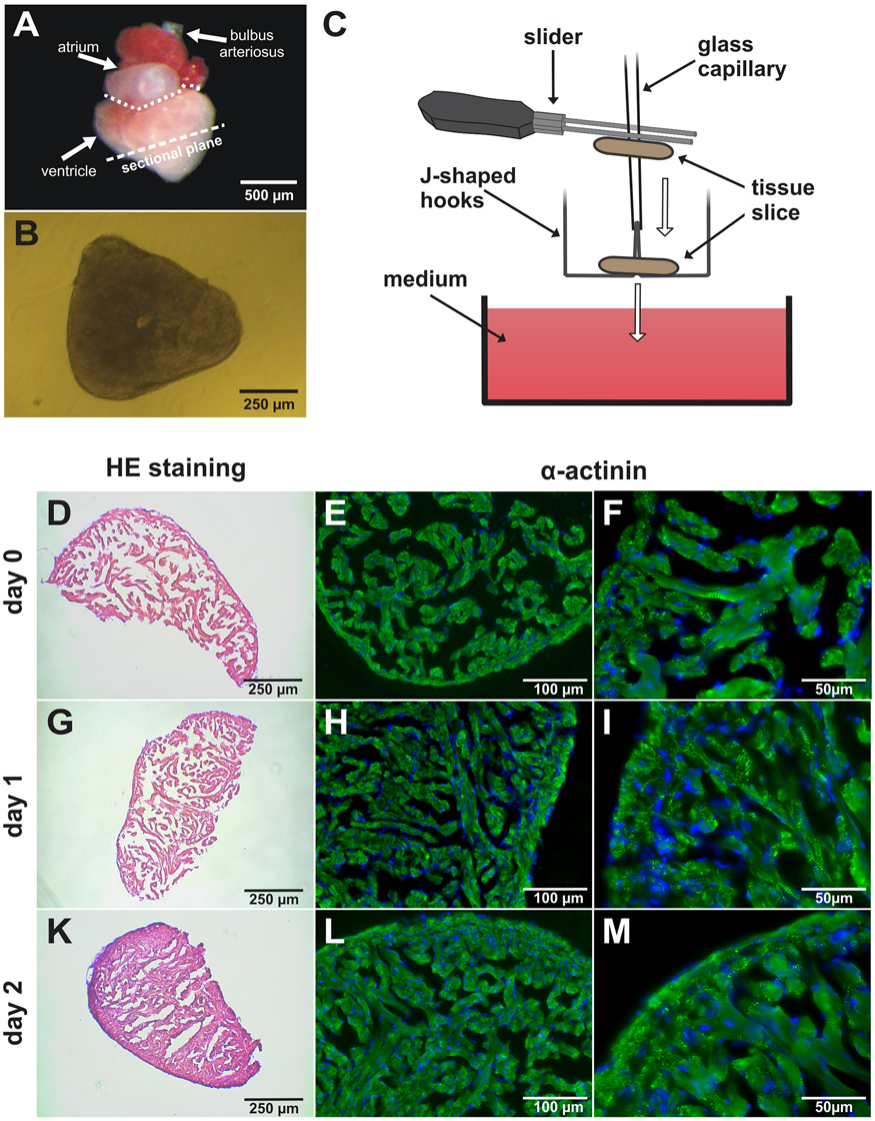
Preparation and histology of zebrafish ventricular myocardial tissue slices. (A) Excised zebrafish heart. Slices were sectioned along the short axis as displayed by the sectional plane. (B) Picture of a myocardial tissue slice. Dashed circle encloses the ventricular cavity used for mounting on the force setup. (C) Technique used for mounting of tissue slices. The slice is impaled on a glass capillary with a conic tip. The lumen of the capillary is placed on the tips of the J-shaped hooks and the tissue slice is pushed down. Afterwards the hooks with the slice are lowered into the medium-containing measuring chamber. (D-M) Histological and immunohistochemical stainings of myocardial tissue slices. HE staining of cryosections (8 μm) obtained from a myocardial tissue slice at day 0 (D), day 1 (G) and day 2 (K). Cyrosections stained against sarcomeric-α-actinin (green) at day 0 (E-F), day 1 (H-I) and day 2 (L-M). Nuclei are counterstained with Hoechst (blue).

Zebrafish were anaesthetised (150 mg/l tricaine in 300 mg/l sodium bicarbonate solution), cooled down on ice and decapitated. Hearts were excised and transferred into cold (4°C) Ca^2+^-free Tyrode’s solution (composition in mM: NaCl 136, KCl 5.4, NaH_2_PO_4_ 0.33, MgCl_2_ 1, glucose 10, HEPES 5; pH 7.4 adjusted with NaOH) continuously bubbled with pure oxygen. Ventricles were separated from the atria and surrounding tissue (Fig 1A) and ventricular slices were prepared as described previously [14]. Briefly, ventricles were embedded in 4% low-melting agarose (Carl Roth, Karlsruhe, Germany). After solidification of the agarose, ventricles were sectioned with steel blades (Campden Instruments, Leicester, England) along the short axis into 300 μm thick tissue slices (Fig 1B and 1C) using a vibratome (VT1000S, Leica Microsystems, Wetzlar, Germany). Up to 3 ventricular slices could be prepared from a single heart. To induce Ca^2+^ tolerance, extracellular Ca^2+^ concentration was increased to 0.9 mM in a single step. After 30 minutes, ventricular slices were transferred to cold (4°C) Iscove’s modified Dulbecco’s medium (IMDM, Life Technologies GmbH, Darmstadt, Germany) supplemented with 20% fetal calf serum (FCS, Life Technologies GmbH). Temperature was raised slowly to 29°C for at least 1 h. Slices were used for experiments between day 0 and 2.

Due to the small dimensions of the zebrafish atrium (Fig 1A), preparation of atrial slices was not feasible. Therefore, for the preparation of atrial myocardium, hearts were transferred into ice-cold Ca^2+^-free Tyrode’s solution and surrounding ventricular and bulbar tissue was removed. Analogue to ventricular tissue, Ca^2+^ concentration was increased to 0.9 mM in a single step and atria were transferred into IMDM supplemented with 20% FCS after 30 min. Temperature was raised slowly to 29°C for at least 1 h.

### Isometric force measurements

Our setup was similar to the one used for force measurements of vascular rings [15]. Since slicing the ventricle orthogonal to the long axis resulted in a tissue ring (Fig 1B), the ventricular cavity provided a preformed hole that facilitated the mounting of the specimen. Slices were mounted onto J-shaped steel needles connected to an isometric force transducer (Scientific Instruments, Heidelberg, Germany) as described in detail previously [14] (Fig 1C). Specimens were immersed in a dish filled with IMDM without serum (1.97 mM Ca^2+^) unless otherwise stated. Temperature was maintained at 29°C and solution was continuously bubbled with carbogen (95% O_2_; 5% CO_2_). Field stimulation was performed by silver electrodes (8–24 V, pulse duration 5 ms) connected to a custom-made stimulator. Length of specimens was increased stepwise to the length of maximal force development during continuous stimulation at 1 or 2 Hz. Analogue signals from the force transducer (KG7A; range 0–5 mN, resolution 0.2 N, resonance frequency 250–300 Hz) were amplified with a bridge amplifier (BAM7C; Scientific Instruments). Signals were digitised at a sampling frequency of 1 kHz. For recording and off-line analysis, we used DasyLab version 7.0 (National Instruments, Munich, Germany).

### Experimental protocols and data analysis

To determine the force-frequency relationship (FFR), ventricular slices were paced according to a stimulation protocol of alternating higher and lower frequencies (3.0, 2.0, 4.0, 2.5, 1.0, 3.5, 1.5, 0.5, 6.0 Hz for 2 minutes at each frequency). The last 30 seconds of each stimulation period were analysed to evaluate the effect of frequency on the twitches. Post-pause potentiation behaviour of the slices was studied by interrupting field stimulation for 5 or 10 seconds after 2 minutes of constant electrical stimulation at 2 Hz. The first beat after quiescent periods was analysed and normalised to the steady-state condition represented by the mean of the developed force of the last 10 beats before the rest interval. Measurements to assess the response to ascending extracellular calcium concentration ([Ca^2+^]_ec_) were performed in Tyrode’s solution allowing the exposure to low [Ca^2+^]_ec_ beneath 1.97 mM. Composition of Tyrode’s solution was equal to the one used for slicing except for the addition of 2 mM sodium pyruvate. All pharmacological experiments were performed during constant field stimulation at 1 or 2 Hz. After recording of baseline conditions for 10 minutes, drugs were administered and recorded for 10 minutes for each concentration. For data analysis, signals were filtered (moving average, high-, and low-pass Bessel filters; 0.05 Hz, 15 Hz, 8^th^ order) and baseline correction was achieved by an automated algorithm (moving arithmetic average of 1000 samples after above described high- and low-pass filtering). To investigate changes of twitch morphology, single contractions were averaged during steady state conditions (30 to 300 seconds before next experimental step) using an automated algorithm.

### Histology

Slices were fixed in 4% paraformaldehyde for 2 hours at room temperature. Afterwards 8 μm thick cyrosections were prepared and stained with hematoxylin & eosin (HE; Carl Roth, Karlsruhe, Germany). Pictures were acquired with a Zeiss Axiovert 200 microscope (Carl Zeiss, Jena, Germany). For immunohistochemical investigations, enzymatic antigen retrieval was carried out by using trypsin. Sarcomeric-α-actinin antibody (clone EA53, 1:800; Sigma-Aldrich) was applied to examine sarcomere morphology. Anti-mouse IgG1 Alexa Fluor 555 (1:1000; Life Technologies GmbH) was used for secondary detection followed by nuclear staining with Hoechst 33342. Stained sections were imaged using a Zeiss Axiovert 200 microscope and the image processing software AxioVision release version 4.6 (both Carl Zeiss).

### Solutions

All chemicals for buffer and stock solution preparation were purchased from Sigma-Aldrich Chemie GmbH, Steinheim, Germany, unless otherwise stated. CaCl_2_ stock solution was dissolved in Tyrode’s solution at a concentration of 100 mM. NiCl_2_ was dissolved in IMDM at a concentration of 200 mM. (±)-Verapamil (VER) stock solution was prepared in demineralised water at a concentration of 1 mM. Ryanodine (Ry) stock solution was prepared in ethanol at a concentration of 2 mM. (-)-Isoproterenol (ISO) was dissolved in Aqua dest. at a concentration of 1 mM with 10 mM ascorbic acid.

### Statistical analysis

Results from pooled data are presented as mean ± SEM. Statistical analysis was performed using an ANOVA test followed by a Student-Newman-Keuls test, a Student t-test or ANOVA repeated measures, as appropriate, using IBM SPSS Statistics Version 22 (IBM, Armonk, NY, USA). p < 0.05 was considered statistically significant.

## Results

### Histology and immunohistochemistry of fresh and cultured slices

The trabecularised structure as well as the cross-striated pattern of α-actinin of the zebrafish ventricle was well preserved after the preparation of ventricular slices (Fig 1D, 1E and 1F). The cross-striated pattern of a-actinin could be observed on day 1 (Fig 1G, 1H and 1I), day 2 (Fig 1K, 1L and 1M) and day 4 days (S1A, S1B and S1C Fig). The trabecularisation was partly lost over the culture period of 4 days (S1A and S1B Fig). When cultured under hypoxic conditions (1% O_2_, 5% CO_2_, 37°C), cross-striation was almost completely lost after 2 days (S1D, S1E and S1F Fig).

### Force frequency relationship

**Fig 2:**
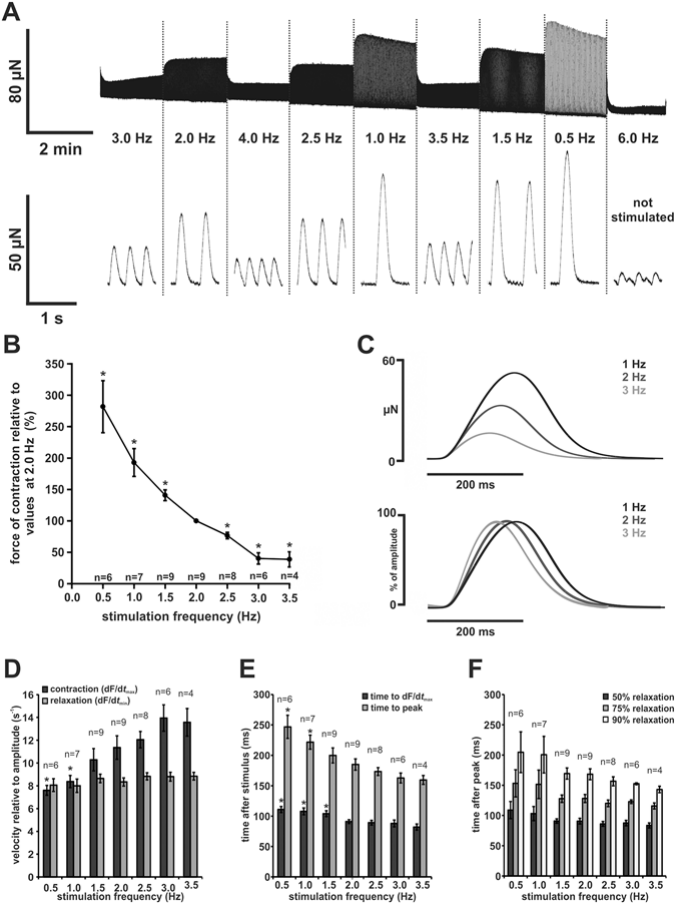
Force-frequency relationship in zebrafish ventricular myocardium. (A) Isometric contractions of a zebrafish ventricular slice (FFR protocol). Original trace (top) and magnified twitches (bottom). Twitch amplitudes were highest at the lowest stimulation frequency (0.5 Hz) and decreased steadily with increasing stimulation frequency. (B) Zebrafish ventricular slices showed an overall negative FFR. Changes of force of contraction were normalised to values at 2 Hz for each individual measurement. (C) Contraction and relaxation kinetics were dependent on the stimulation frequency (representative experiment, top: original averaged twitches, bottom: normalised averaged twitches). Peak force was reached faster at higher stimulation frequencies. (D) Maximal contraction velocity normalised to amplitude (dF/d*t*_max_) was increased at higher stimulation rates, while maximal relaxation velocity normalised to amplitude (dF/d*t*_min_) was unchanged. (E) Time to point of maximal contraction velocity (dF/d*t*_max_) and time to peak (TTP) decreases at higher stimulation rates indicating an acceleration of contraction. (F) Relaxation of ventricular slices was also accelerated with increasing stimulation frequency, but not as marked as during contraction. All data are expressed as mean ± SEM; asterisks indicate statistical significant differences (p < 0.05 vs. values at a stimulation frequency of 2 Hz).

We observed an overall negative FFR (Fig 2A and 2B) in all preparations. Stimulation frequencies in our protocol ranged from 0.5 Hz to 6 Hz, however none of the preparations was able to follow stimulation frequencies of 6 Hz and only 1 preparation reached 4 Hz. 4/9 preparations were able to contract at 3.5 Hz, 6/9 at 3 Hz, 8/9 at 2.5 Hz, and 9/9 at both 1.0 Hz and 1.5 Hz. Due to spontaneous contractions, stimulation rates of 1.0 Hz and 0.5 Hz could be achieved only in 7/9 and 6/9 preparations, respectively. Force increased at lower frequencies (0.5 Hz: 282 ± 41%, n = 6; 1.0 Hz: 193 ± 22%, n = 7; 1.5 Hz: 140 ± 9%, n = 9, all p < 0.05, normalised to force of contraction at 2 Hz of individual experiments) and decreased at higher frequencies (2.5 Hz: 77 ± 5%, n = 8; 3.0 Hz: 40 ± 9 Hz, n = 6; 3.5 Hz: 39 ± 12, n = 4, all p < 0.05, normalised to force of contraction at 2 Hz).

As changes in resting tension might contribute to a negative FFR, we calculated the ratio of change in resting tension and change in amplitude in order to express the contribution of changes in resting tension to a negative FFR. If frequency was reduced from 2 Hz to 1 Hz, a decrease in resting tension contributed to 16 ± 5% of amplitude change, if increased from 1 Hz to 3 Hz, an increase in resting tension contributed to amplitude change by 6 ± 1%. If decreased from 3 Hz to 2 Hz, resting tension contributed 17 ± 4% of amplitude change (S2 Fig).

Twitch morphology was strongly affected by changes in contraction frequency (Fig 2C). Contraction as well as relaxation accelerated at higher frequencies. Maximum contraction velocity relative to twitch amplitude increased from 7.6 ± 0.4 s^-1^ at 0.5 Hz to 13.6 ± 1.2 s^-1^ at 3.5 Hz (Fig 2D). Correspondingly, time to peak gradually decreased from 247 ± 19 ms at 0.5 Hz (n = 6) to 160 ± 7 ms at 3.5 Hz (n = 4) (Fig 2E). Duration of relaxation shortened with increasing frequencies indicated by the decrease of time after peak to 50%, 75%, and 90% relaxation (50% relaxation: 109 ± 14 ms at 0.5 Hz to 84 ± 4 ms at 3.5 Hz; 75% relaxation: 153 ± 23 ms at 0.5 Hz to 116 ± 5 ms at 3.5 Hz; 90% relaxation: 205 ± 34 ms at 0.5 Hz to 143 ± 4 ms at 3.5 Hz, n = 6 for 0.5 Hz and n = 4 for 3.5 Hz) (Fig 2F).

### Effects of [Ca^2+^]_ec_ and LTCC inhibition

**Fig 3:**
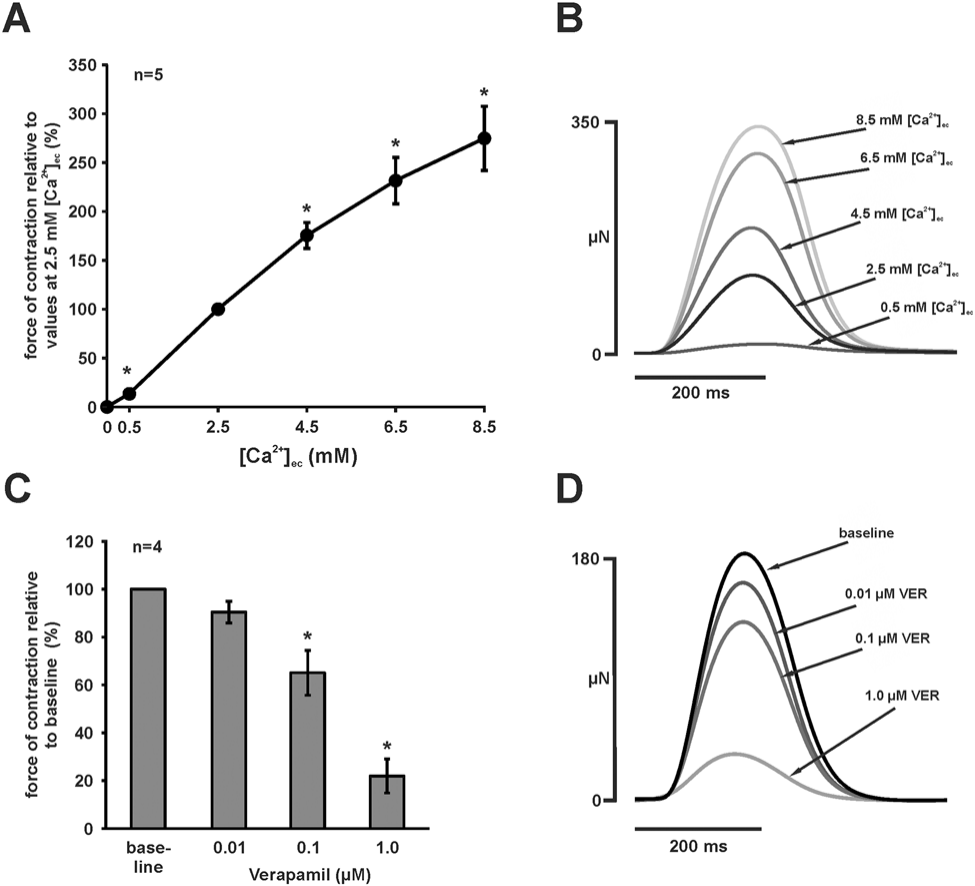
Influence of extracellular calcium concentration and LTCC inhibition. (A) Higher [Ca^2+^]_ec_ led to increased force of contraction (constant field stimulation, 1 or 2 Hz). In nominally Ca^2+^-free Tyrode’s solution, no force was generated. Force of contraction for each [Ca^2+^]_ec_ was normalised to values at 2.5 mM [Ca^2+^]_ec_. Results are expressed as mean ± SEM, asterisks indicate significant differences to force of contraction at 2.5 mM [Ca^2+^]_ec_ (p < 0.05). (B) Effect of ascending [Ca^2+^]_ec_ on twitch morphology (representative experiment, averaged twitches). Twitches were mildly prolonged for high [Ca^2+^]_ec_. (C) Administration of LTCC blocker VER reduced force of contraction in a dose-dependent manner. Data are expressed as mean normalised to baseline ± SEM; asterisks indicate statistically significant differences (p < 0.05 vs. baseline). (D) Original averaged twitches of a representative experiment showing the twitch amplitude reduction after VER administration.

Myocardial contractility strongly depends on [Ca^2+^]_ec_. To test the effects of varying [Ca^2+^]_ec_ on force of contraction, we increased [Ca^2+^]_ec_ stepwise (0.0, 0.5, 2.5, 4.5, 6.5, 8.5 mM) using a nominally Ca^2+^-free Tyrode’s solution under constant field stimulation at 1–2 Hz. Withdrawal of extracellular Ca^2+^ abolished generation of force completely in all preparations (n = 5). The force of contraction increased with ascending [Ca^2+^]_ec_ (Fig 3A and 3B). Compared to force of contraction at [Ca^2+^]_ec_ of 2.5 mM, force of contraction was 13 ± 3% at 0.5 mM and increased to 175 ± 13% at 4.5 mM, to 231 ± 24 at 6.5 mM, and to 275 ± 33% at 8.5 mM (n = 5, all p < 0.05 compared to [Ca^2+^]_ec_ of 2.5 mM).

Application of the LTCC blocker VER decreased force of contraction in a dose-dependent manner (0.01 μM: 90 ± 4%, 0.1 μM: 65 ± 9%, 1 μM: 22 ± 7%, compared to baseline, n = 4, p < 0.05 for baseline vs. 0.1 μM and 1 μM) (Fig 3C). Twitch morphology was not affected by application of VER (Fig 3D).

### Post pause potentiation

**Fig 4:**
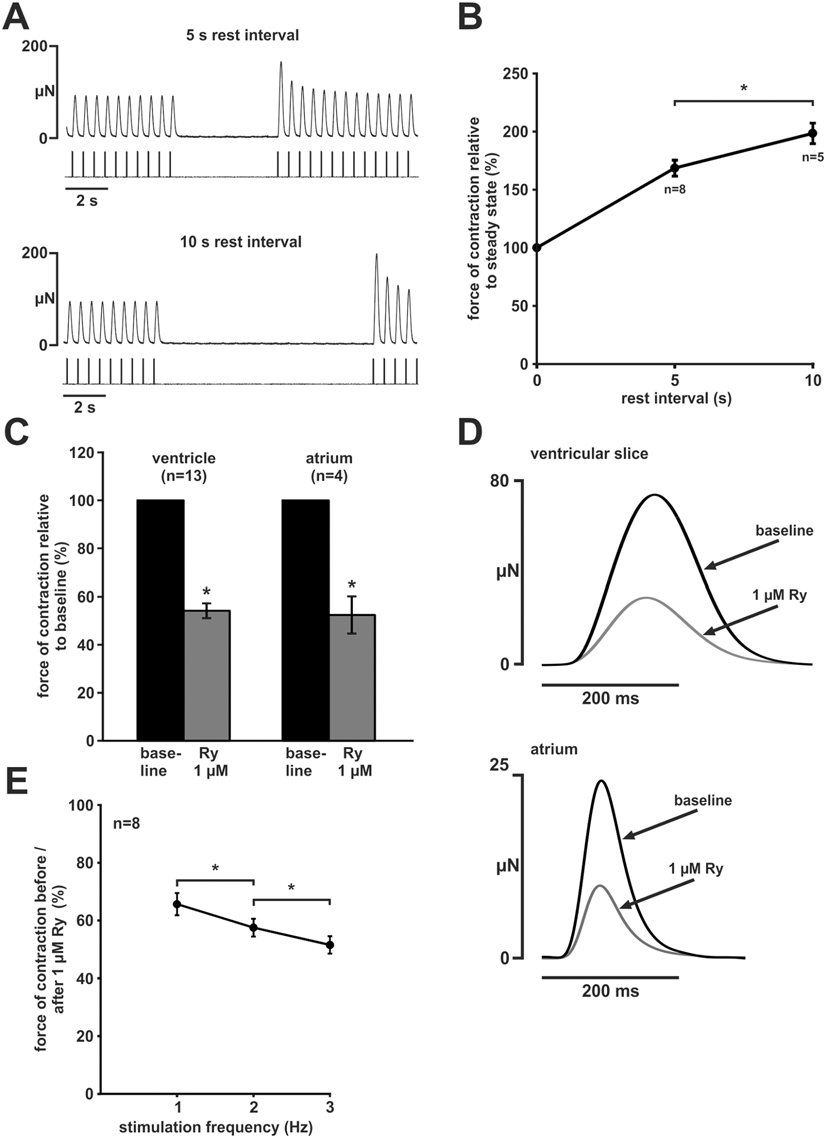
Contribution of SR to developed force. (A) Post-pause potentiation behaviour of zebrafish ventricular slices (representative experiment, top: 5 seconds rest-interval, bottom: 10 seconds rest-interval). (B) The twitch amplitudes of the first contraction were increased after a rest interval. Prolonging of rest intervals resulted in higher amplitudes. Force of contraction was normalised to steady-state force before the pause. (C) Effect of Ry Blocking of RyR receptor by 1 μM Ry leads to a reduction of force of contraction in ventricular slices and whole atrium. Force of contraction was normalised to baseline. (D) Original averaged twitches before and after Ry application of a ventricular slice (upper panel) and an atrium (lower panel). (E) Abbreviated FFR protocol (2.0Hz, 1.0 Hz, 3.0 Hz) before and after application of Ry (1 μM). Force of contraction decreased stronger at higher stimulation frequencies after application of Ry. Data are expressed as mean ±SEM, asterisks indicate statistically significant differences (p < 0.05 vs. steady state or between stimulation frequencies).

A main feature of mammalian myocardium is an increase in contractility after rest which—in mammals—mainly depends on intact SR Ca^2+^ loading during rest. We studied post pause potentiation after 5 s and 10 s (Fig 4A and 4B) rest intervals. Force of contraction increased to 169 ± 7% after 5 s rest compared to steady state baseline (n = 8, p < 0.05) and 198 ± 9% after 10 s rest compared to steady state baseline (n = 5, p < 0.05). Thus, we observed a considerable degree of post pause potentiation.

### Inhibition of RyR mediated SR Ca^2+^-release

As post pause potentiation suggests an involvement of SR Ca^2+^-cycling in force generation, we further characterised the relevance of SR Ca^2+^-release by inhibition of RyR by Ry (1 μM). Ry decreased force of contraction to 54 ± 3% (n = 13, p < 0.05) compared to baseline (Fig 4C and 4D). Twitch morphology was not affected by Ry (Fig 4D).

As SR Ca^2+^-cycling is involved in the frequency-dependent regulation of force, we postulated that Ry would affect FFR in zebrafish myocardium. We used an abbreviated FFR protocol (2.0 Hz, 1.0 Hz, 3.0 Hz, 2 minutes each) before and after application of Ry (Fig 4E). Compared to baseline, force of contraction decreased significantly to 66 ± 4% at 1 Hz, 58 ± 3% at 2 Hz, and 52 ± 3% at 3 Hz (n = 8, p < 0.05 each). Thus, contribution of SR Ca^2+^-release to force generation seems to be frequency-dependent.

In addition to ventricular myocardium, we postulated that SR Ca^2+^-release contributes to force generation in the zebrafish atrium. While atrial myocardium showed a higher maximum contraction and relaxation velocity (maximum contraction velocity normalised to twitch amplitude: atrial (n = 4) 23.4 ± 4.3 s^-1^ vs ventricular (n = 13) 11.8 ± 0.3 s^-1^, p < 0.05; maximum relaxation velocity normalised to twitch amplitude: atrial (n = 4) 15.6 ± 1.5 s^-1^ vs ventricular (n = 13) 10.9 ± 0.3 s^-1^, p < 0.05), application of ryanodine decreased force of contraction to 52 ± 8% (n = 4, p < 0.05) compared to baseline (Fig 4C and 4D) Thus, despite a different twitch morphology, RyR-dependent Ca^2+^-release substantially contributed to force generation both in ventricular and atrial myocardium.

### Effects of NiCl_2_

**Fig 5:**
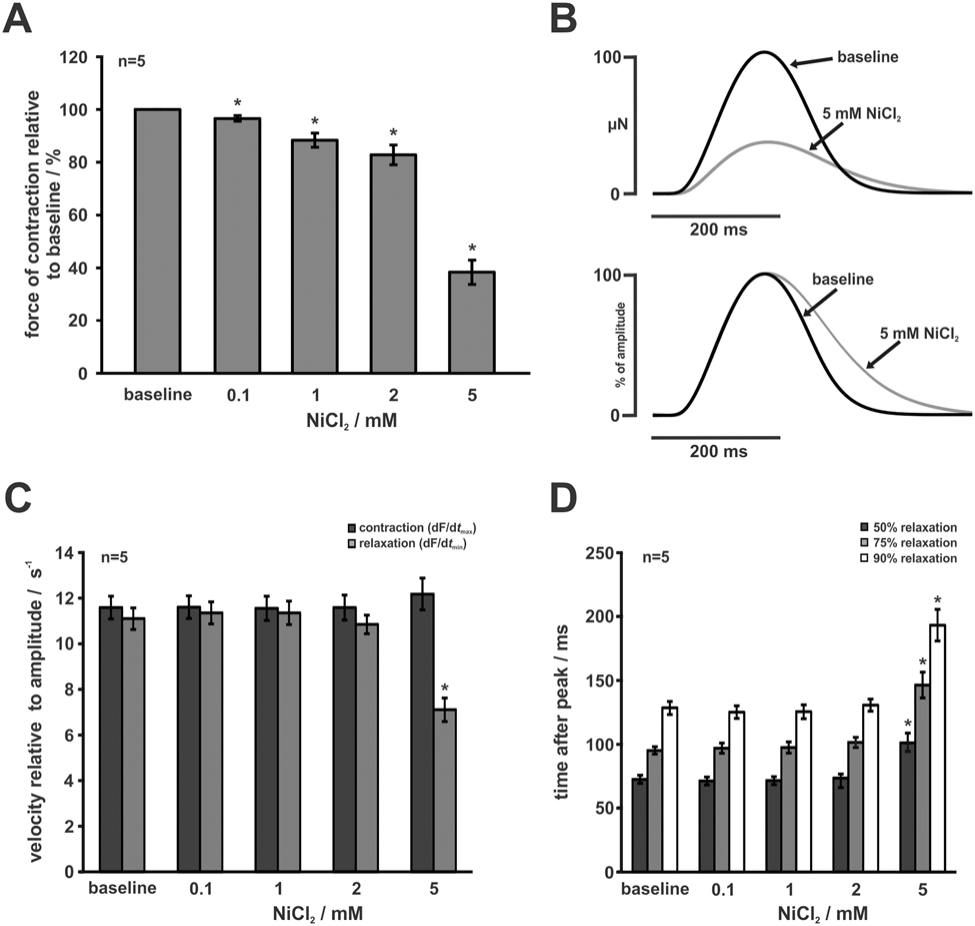
Effects of Ni^2+^. (A) Force of contraction after application of increasing doses of NiCl_2_ relative to baseline. NiCl_2_ reduced developed force in a dose-dependent manner. (B) Averaged twitches of a single experiment after application of 5 mM NiCl_2_ compared to baseline. Upper panel: peak force of contraction was reduced. Lower panel (normalised to baseline): relaxation was prolonged after application of 5 mM NiCl_2_. (C) Contraction and relaxation kinetics relative to amplitude. Maximum contraction velocity (dF/d*t*_max_) was not affected by NiCl_2_. Maximum relaxation velocity (dF/d*t*_min_) was significantly reduced after application of 5 mM NiCl_2_, but not affected at lower concentrations. (D) Time after peak to 50%, 75%, and 90% relaxation after application of NiCl_2_. Only 5 mM, but not lower concentrations, of NiCl_2_ prolonged relaxation significantly. Data are represented as mean ± SEM, asterisks indicate statistically significant differences (* p < 0.05 vs. baseline).

To study the effects of NCX inhibition on force generation, we used NiCl_2_ which is known for its moderately specific inhibitory action on NCX. Application of NiCl_2_ decreased force of contraction to 97 ± 1% at 0.1 mM, 88 ± 3% at 1.0 mM, 83 ± 4% at 2.0 mM and 38 ± 5% at 5.0 mM (n = 5, all p < 0.05 compared to baseline) (Fig 5A). In contrast to the LTCC blocker VER and RyR inhibition by Ry, NiCl_2_ significantly prolonged relaxation at a concentration of 5.0 mM (time after peak to 50% relaxation: 73 ± 3 ms vs. 101 ± 8 ms; 75% relaxation: 95 ± 3 ms vs. 146 ± 10 ms; 90% relaxation: 128 ± 5 vs. 193 ± 12 ms, n = 5, all p < 0.05) (Fig 5B and 5D). Consistently, maximum relaxation velocity relative to twitch amplitude was reduced from 11.1 ± 0.5 s^-1^ to 7.1 ± 0.5 s^-1^ (n = 5, p < 0.05) (Fig 5C). Relaxation was not affected by lower concentrations and maximum contraction velocity was not changed by NiCl_2_ at all (Fig 5C and 5D).

### Effects of β-adrenergic stimulation

**Fig 6:**
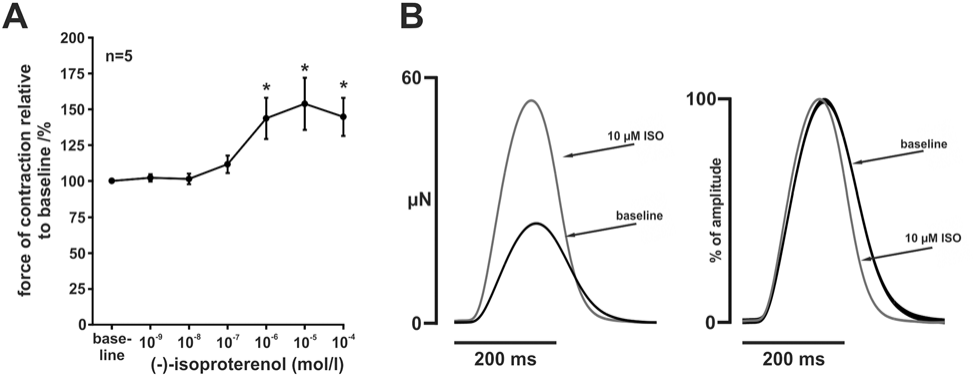
β-adrenergic stimulation. (A) Administration of β-adrenergic agonist ISO resulted in a dose-dependent increase of developed force under constant field stimulation of 2 Hz. Data are expressed as mean ± SEM, asterisks indicate statistically significant differences (p < 0.05 vs. baseline). (B) Original averaged twitches (left) before and after administration of 10 μM ISO and the corresponding normalised averaged twitches (right). Relaxation was mildly accelerated.

The β-adrenergic agonist ISO increased force of contraction (Fig 6A and 6B). Normalised to baseline, force of contraction increased to 102 ± 3% at ISO 10^-9^ M, to 101 ± 4% at ISO 10^-8^ M, to 112 ± 6% at ISO 10^-7^ M, 144 ± 14% at ISO 10^-6^ M and 154 ± 18% at ISO 10^-5^ M (n = 5, p < 0.05 for baseline vs. ISO 10^-6^ and 10^-5^ M, respectively). Force did not further increase at ISO 10^-4^ M (145 ± 13%, p < 0.05 vs. baseline). In addition to the positive inotropic effect, ISO accelerated relaxation mildly (Fig 6B). Maximum relaxation velocity normalised to twitch amplitude was increased from 12.0 ± 0.5 s^-1^ at baseline to 12.6 ± 0.5 s^-1^ at ISO 10^-7^ M, to 13.5 ± 0.4 s^-1^ at 10^-6^ M, to 14.3 ± 0.3 s^-1^ at 10^-5^ M and to 14.3 ± 0.2 at ISO 10^-4^ M (n = 5; p < 0.05 vs. baseline). Shortening velocity was significantly increased only at ISO 10^-4^ M (12.9 ± 0.6 s^-1^ vs. 12.0 ± 0.5 s^-1^ at baseline, p < 0.05), but not at lower concentrations.

### Effects of temperature changes on contractility

Normalised to 29°C, force of contraction decreased to 88 ± 7% at 23°C and increased to 108 ± 6% at 32°C and to 129 ± 11% at 37°C (n = 5, n.s., S3A and S3B Fig). Contraction as well as relaxation velocity were significantly decreased at 23°C and increased at 32°C and 37°C (Contraction velocity relative to amplitude: 8.4 ± 0.7 s^-1^ at 23°C, 14.5 ± 1.0 s^-1^ at 32°C and 21.3 ± 2.0 s^-1^ at 37°C vs. 11.7 ± 0.8 s^-1^ at 29°C, n = 5, p < 0.05; relaxation velocity relative to amplitude: 8.5 ± 0.2 s^-1^ at 23°C, 12.8 ± 0.3 s^-1^ at 32°C and 14.9 ± 0.6 s^-1^ at 37°C vs. 11.6 ± 0.2 s^-1^ at 29°C, n = 5, p < 0.05; S3B and S3C Fig). Accordingly, relaxation kinetics were significantly affected (S3D Fig).

## Discussion

In the present study, we used isometric force measurements of zebrafish ventricular heart slices in order to characterise intrinsic and extrinsic regulators of force of contraction and to determine whether zebrafish ventricular myocardium has similar contractile properties to mammalian myocardium. In summary, we observed that (1) viable slices of zebrafish ventricular myocardium could be prepared, (2) zebrafish ventricular myocardium showed an overall-negative FFR, (3) contractility was regulated by extracellular Ca^2+^ and trans-sarcolemmal Ca^2+^-flux via LTCC, (4) application of NiCl_2_ but not VER prolonged relaxation, and most importantly, (5) inhibition of SR Ca^2+^-release by application of Ry significantly reduced force of contraction.

Due to the simplicity of the preparation and experimental approach, heart slices have become a popular technique especially in the recent years and have been prepared from many mammalian species [16]. While a zebrafish whole-heart preparation is relatively straightforward compared to mammalian whole-heart preparations and can even be maintained in culture up to three days in vitro [17], heart slices are particularly suitable for isometric force measurements of ventricular working myocardium [14]. Histologically and immunohistochemically, we observed a preservation of the sarcomeric organisation of α-actinin up to four days after preparation. Trabecularisation was well preserved in the fresh ventricular slice; after prolonged culture, a slight compaction could be noted. Thus, structure seems to be well preserved after slicing. If aiming at long-term culture, culture conditions need to be further optimised.

We observed an overall negative FFR (Fig 2A). This is in line with other fish species which show an overall or secondary-phase negative FFR [18] and in contrast with the majority of mammalian species and preparations [12]. Indeed, a negative FFR is generally considered a feature of human heart disease, e.g. dilated cardiomyopathy [19]. To our knowledge, a positive FFR which is commonly seen in adult mammalian myocardium has not yet been reported in teleost fish hearts [18]. It is therefore assumed that cardiac output is regulated mainly via changes in stroke volume only rather than heart rate [20].

In adult mammalian cardiomyocytes, intact SR Ca^2+^ cycling and mechanical reconstitution are generally considered a requirement for a positive FFR [21]. In our preparation, calculation of the ratio between changes in resting tension and changes in amplitude (S2 Fig) shows that incomplete mechanical reconstitution contributes to negativity of the FFR to a small extent. As, however, EC coupling relies on trans-sarcolemmal Ca^2+^-influx, frequency-dependent reduction of trans-sarcolemmal Ca^2+^-influx can be an alternative explanation for a negative FFR. Consistent with this idea, it has been shown that LTCC current decreases at higher frequencies in zebrafish ventricular cardiomyocytes [13]. Thus, a negative FFR does not necessarily rule out a significant contribution of SR Ca^2+^-release to EC coupling.

In order to further characterise the role of several components of EC coupling, we used specific pharmacological interventions including changes of [Ca^2+^]_ec_ and the LTCC blocker VER to modify trans-sarcolemmal Ca^2+^-flux, Ry to inhibit SR Ca^2+^-release, ISO to stimulate β-adrenergic effects on inotropy and lusitropy, and NiCl_2_ to inhibit NCX. Following this order, we discuss the effects of pharmacological interventions and putative mechanisms in the following paragraphs.

While it was not suprising that force of contraction was strongly influenced by varying [Ca^2+^]_ec_ and by the LTCC inhibitor verapamil, we were surprised by a ~50% reduction of force both in atrial and ventricular myocardium after application of Ry. This points to an important role of SR Ca^2+^-release in zebrafish myocardium. The role of SR Ca^2+^-release in EC coupling of teleost fish is controversial as data are highly dependent on species and experimental conditions [22]. Recently, Bovo *et al*. reported that SR Ca^2+^-release contributes less than 20% of the total Ca^2+^-transient and that RyR expression is at least 72% lower in isolated zebrafish than in rabbit ventricular cardiomyocytes [4]. Interestingly though, in the same study, a significant amount of Ca^2+^ was released from the SR after application of caffeine. A high SR Ca^2+^-load has also been found in other teleost species including rainbow trout [23] and seems to be generally higher in fish than in mammals [22]. In isolated ventricular trabeculae of rainbow trout, SR Ca^2+^-release significantly contributes to force development at 22°C to a higher extent than at 12°C [24]. In the present study, we observed a significant decrease of peak force (≈ 50%) after application of 1 μM Ry. This shows that EC coupling is highly dependent on SR Ca^2+^-release under our experimental conditions. This is further supported by the finding that zebrafish ventricular myocardium showed a strong post pause potentiation already after 5–10 seconds. Interestingly, inhibition of SR Ca^2+^-release reduced force of contraction stronger at higher frequencies. Thus, FFR became more negative with inhibition of SR Ca^2+^-release. Although this finding is difficult to interpret given potential frequency-dependent changes in myofilament sensitivity to cytosolic Ca^2+^-concentrations, and a potential use-dependence of RyR inhibition [25], one might speculate that SR Ca^2+^-release is increased in order to compensate frequency-dependent inhibition of LTCC. This would further underline the important role of SR function in zebrafish ventricular myocardium.

In our study, and other published work in the zebrafish [26], contraction and relaxation kinetics were strongly influenced by varying temperature (S3 Fig). Bearing in mind that Ca^2+^-cycling and especially SR function strongly depend on temperature in the fish heart [24], we chose experimental temperature (29°C) close to housing temperatures (27.2°C). Therefore, we assume that SR Ca^2+^-release contributes to EC coupling *in vivo* at least under laboratory housing conditions.

In further support of the existence of an intact SR Ca^2+^-cycling, we observed a mildly positive lusitropic effect of the β-adrenergic agonist ISO in addition to a positive inotropic response (Fig 6B). This could potentially be explained by phosphorylation of phospholamban and subsequent disinhibition of the SR Ca^2+^-ATPase, SERCA [27]. However, NCX phosphorylation due to β-adrenergic stimulation has also been reported to accelerate relaxation [28] and might be an alternative explanation. On the contrary, ISO decreases NCX current in the frog ventricle [29] and could therefore blunt the positive lusitropic effect of SERCA disinhibition. As it is unknown, whether NCX current is reduced, enhanced, or unchanged by β-adrenergic stimulation in the zebrafish ventricle, this finding needs to be interpreted with caution.

The importance of NCX for myocardial relaxation, however, has been firmly established for many species and preparations [12], although zebrafish cardiac NCX has—to our knowledge—not been functionally studied using the patch-clamp technique. Bovo et al. [4] could show that Ca^2+^ decline after caffeine application was slowed down in Na^+^/Ca^2+^-free solution which indicates that NCX is an important factor in relaxation of zebrafish ventricular cardiomyocytes. In the present study, we used NiCl_2_ which is known to block NCX at higher concentrations, but also exerts a dose-dependent inhibitory action on Ca^2+^ channels [30]. Ni^2+^ reduced force of contraction in a dose dependent manner and prolonged relaxation at a high concentration (5 mM), but not at lower concentrations. While the dose-dependent reduction of force of contraction might also underlie additional inhibition of LTCC and T-type calcium currents, prolongation of relaxation can only be explained by the inhibitory action of Ni^2+^ on NCX which occurs at high concentrations (5–10 mM) [30]. In support of this, application of VER which seems to block not only LTCC, but also T-type calcium channels [31], does not affect relaxation (Fig 3D). We can therefore conclude that the Ni^2+^-induced deceleration of relaxation is mainly caused by NCX inhibition, and that NCX significantly contributes to Ca^2+^-extrusion under our experimental conditions.

## Limitations

In the present study, force data can only be compared between measurements of the same preparation, but not between different preparations as force was not normalised to the cross-sectional area of the preparation. However, as fibre orientation in the zebrafish ventricle is not aligned to a specific axis but non-parallel, a large proportion of fibres will be oriented with an unknown angle to the axis of the measurement setup. Therefore, we avoided normalisation to the cross-sectional area as this might give false impression of comparability between measurements in different preparations. As our main aim was to study the role of different force regulatory mechanisms but not to give absolute values of generated force, this limitation does not interfere with the aims of the study.

The main finding of our study is that SR Ca^2+^-release substantially contributed to force generation in the zebrafish ventricle, although force-frequency relationship was negative. We are not able to explain why the force-frequency relationship is negative in the majority of fish species. It can, however, be assumed that the influences of external variables on the mechanical environment in the fish heart is much more complex than in mammalian hearts. For example, ventricular compliance is influenced by diving depth and temperature as well as the elasticity of the *bulbus arteriosus* and thus, afterload and resonant frequency of the vessel downstream to the heart. To fully understand these complex mechanical dynamics, a completely different experimental or numerical approach is required.

## Conclusions

In conclusion, we observed that despite the overall-negative FFR, regulation of contractile force in zebrafish ventricular myocardium strongly depends on extracellular Ca^2+^, trans-sarcolemmal Ca^2+^ influx, NCX-driven Ca^2+^ extrusion, and SR Ca^2+^-release. Thus, although some regulatory mechanisms of force development seem to be similar between zebrafish and mammals, including SR Ca^2+^-release, one of the most important regulatory principles of contractile force—the relationship between force and frequency—is contrary between the mammalian and the zebrafish heart. Beyond a *per se* interest in fish cardiac physiology, these aspects need to be considered when using zebrafish to model human diseases of myocardial contractility.

## Supporting Information

S1 Fig Characterisation of zebrafish ventricular myocardial slices after prolonged culture and cultured under hypoxic conditions. HE staining of cryosections (8 μm) obtained from a myocardial tissue slice at day 4 (A) and cultured under hypoxic conditions (1% O_2_ for 2 days) (D). Cyrosections stained against sarcomeric-α-actinin (green) at day 4 (B-C) or cultured under hypoxic conditions (E-F). Nuclei are counterstained with Hoechst (blue).
(TIF)

S2 Fig Contribution of resting tension changes to negative FFR. Box plots of the ratio of change in resting tension and change in amplitude after frequency switches. If frequency was reduced from 2 Hz to 1 Hz, a decrease in resting tension contributed to 16± 5% of amplitude increase, if increased from 1 Hz to 3 Hz, an increase in resting tension contributed to amplitude change by 6 ± 1%, and if decreased from 3 Hz to 2 Hz, resting tension contributed to 17 ± 4%.
(TIF)

S3 Fig Effects of temperature. (A) Force of contraction increased with elevated temperature. Force of contraction was normalized to values at 29°C. Results are expressed as mean ± SEM (B) Original averaged twitches of a representative experiment showing the influence of investigated temperatures on twitch amplitude as well as contraction and relaxation kinetics. (C) Maximal contraction (dF/d*t*_max_) and relaxation (dF/d*t*_min_) velocities normalized to amplitude increased with temperature. (D) Relaxation of the myocardial slices was also accelerated at elevated temperatures. All data are expressed as mean ± SEM; asterisks indicate statistical significant differences (p < 0.05 vs. values at 29°C).
(TIF)

## References

[1] Bakkers J. Zebrafish as a model to study cardiac development and human cardiac disease. Cardiovasc Res. 2011; 91: 279–288. 10.1093/cvr/cvr098 21602174 PMC3125074

[2] Hu N, Yost HJ, Clark EB. Cardiac morphology and blood pressure in the adult zebrafish. Anat Rec. 2001; 264: 1–12. 11505366 10.1002/ar.1111

[3] Brette F, Luxan G, Cros C, Dixey H, Wilson C, Shiels HA. Characterization of isolated ventricular myocytes from adult zebrafish (Danio rerio). Biochem Biophys Res Commun. 2008; 374: 143–146. 10.1016/j.bbrc.2008.06.109 18602892 PMC2581121

[4] Bovo E, Dvornikov AV, Mazurek SR, de Tombe PP, Zima AV. Mechanisms of Ca^2^+ handling in zebrafish ventricular myocytes. Pflugers Arch. 2013; 465: 1775–1784. 10.1007/s00424-013-1312-2 23821298 PMC4138713

[5] Tessadori F, van Weerd JH, Burkhard SB, Verkerk AO, de Pater E, Boukens BJ, et al Identification and functional characterization of cardiac pacemaker cells in zebrafish. PLoS ONE. 2012; 7: e47644 10.1371/journal.pone.0047644 23077655 PMC3473062

[6] Barrionuevo WR, Burggren WW. O_2_ consumption and heart rate in developing zebrafish (Danio rerio): influence of temperature and ambient O_2_ . Am J Physiol. 1999; 276: 505–513. 10.1152/ajpregu.1999.276.2.R505 9950931

[7] Arnaout R, Ferrer T, Huisken J, Spitzer K, Stainier DY, Tristani-Firouzi M, et al Zebrafish model for human long QT syndrome. Proc Natl Acad Sci U S A. 2007; 104: 11316–11321. 17592134 10.1073/pnas.0702724104 PMC2040896

[8] Xu X, Meiler SE, Zhong TP, Mohideen M, Crossley DA, Burggren WW, et al Cardiomyopathy in zebrafish due to mutation in an alternatively spliced exon of titin. Nat Genet. 2002; 30: 205–209. 11788825 10.1038/ng816

[9] Zhao L, Zhao X, Tian T, Lu Q, Skrbo-Larssen N, Wu D, et al Heart-specific isoform of tropomyosin4 is essential for heartbeat in zebrafish embryos. Cardiovasc Res. 2008; 80: 200–208. 10.1093/cvr/cvn177 18583338

[10] Sehnert AJ, Huq A, Weinstein BM, Walker C, Fishman M, Stainier DY. Cardiac troponin T is essential in sarcomere assembly and cardiac contractility. Nat Genet. 2002; 31: 106–110. 11967535 10.1038/ng875

[11] Rottbauer W, Wessels G, Dahme T, Just S, Trano N, Hassel D, et al Cardiac myosin light chain-2: a novel essential component of thick-myofilament assembly and contractility of the heart. Circ Res. 2006; 99: 323–331. 16809551 10.1161/01.RES.0000234807.16034.fe

[12] Bers DM. Excitation-contraction coupling and cardiac contractile force. 2nd ed. Dordrecht: Kluwer Academic Publishers; 2001.

[13] Zhang PC, Llach A, Sheng XY, Hove-Madsen L, Tibbits GF. Calcium handling in zebrafish ventricular myocytes. Am J Physiol Regul Integr Comp Physiol. 2011; 300: R56–R66. 10.1152/ajpregu.00377.2010 20926764

[14] Pillekamp F, Halbach M, Reppel M, Rubenchyk O, Pfannkuche K, Xi JY, et al Neonatal murine heart slices. A robust model to study ventricular isometric contractions. Cell Physiol Biochem. 2007; 20: 837–846. 17982265 10.1159/000110443

[15] Angus JA, Wright CE. Techniques to study the pharmacodynamics of isolated large and small blood vessels. J Pharmacol Toxicol Methods. 2000; 44: 395–407. 11325582 10.1016/s1056-8719(00)00121-0

[16] Wang K, Terrar D, Gavaghan DJ, Mu-U-Min R, Kohl P, Bollensdorff C. Living cardiac tissue slices: an organotypic pseudo two-dimensional model for cardiac biophysics research. Prog Biophys Mol Biol. 2014; 115:314–327. 10.1016/j.pbiomolbio.2014.08.006 25124067

[17] Pieperhoff S, Wilson KS, Baily J, de Mora K, Maqsood S, Vass S, et al Heart on a Plate: Histological and Functional Assessment of Isolated Adult Zebrafish Hearts Maintained in Culture. PLoS ONE. 2014; 9: e96771 10.1371/journal.pone.0096771 24824845 PMC4019527

[18] Shiels HA, Vornanen M, Farrell AP. The force-frequency relationship in fish hearts-a review. Comp Biochem Physiol A Mol Integr Physiol. 2002; 132: 811–826. 12095865 10.1016/s1095-6433(02)00050-8

[19] Schwinger RH, Böhm M, Erdmann E. Inotropic and lusitropic dysfunction in myocardium from patients with dilated cardiomyopathy. Am Heart J. 1992; 123:116–128. 1309621 10.1016/0002-8703(92)90755-k

[20] Shiels HA, Calaghan SC, White E. The cellular basis for enhanced volume-modulated cardiac output in fish hearts. J Gen Physiol. 2006; 128:37–44. 16769795 10.1085/jgp.200609543 PMC2151555

[21] Endoh M. Force-frequency relationship in intact mammalian ventricular myocardium: physiological and pathophysiological relevance. Eur J Pharmacol. 2004; 500: 73–86. 15464022 10.1016/j.ejphar.2004.07.013

[22] Haverinen J, Vornanen M. Comparison of sarcoplasmic reticulum calcium content in atrial and ventricular myocytes of three fish species. Am J Physiol Regul Integr Comp Physiol. 2009; 297: R1180–1187. 10.1152/ajpregu.00022.2009 19692664

[23] Hove-Madsen L, Llach A, Tort L. Quantification of Ca^2+^ uptake in the sarcoplasmic reticulum of trout ventricular myocytes. Am J Physiol. 1998; 275: R2070–2080. 9843899 10.1152/ajpregu.1998.275.6.R2070

[24] Shiels H, Farrell A. The effect of temperature and adrenaline on the relative importance of the sarcoplasmic reticulum in contributing Ca^2+^ to force development in isolated ventricular trabeculae from rainbow trout. J Exp Biol. 1997; 200: 1607–1621. 9319512 10.1242/jeb.200.11.1607

[25] Malecot CO, Katzung BG. Use-dependence of ryanodine effects on postrest contraction in ferret cardiac muscle. Circ Res. 1987; 60:560–567. 3594740 10.1161/01.res.60.4.560

[26] Denvir MA, Tucker CS, Mullins JJ. Systolic and diastolic ventricular function in zebrafish embryos: influence of norepenephrine, MS-222 and temperature. BMC Biotechnol. 2008; 8:21 10.1186/1472-6750-8-21 18304347 PMC2291041

[27] Tada M, Katz AM. Phosphorylation of the sarcoplasmic reticulum and sarcolemma. Annu Rev Physiol. 1982; 44: 401–423. 6280588 10.1146/annurev.ph.44.030182.002153

[28] Han X, Ferrier GR. Contribution of Na^+^-Ca^2+^ exchange to stimulation of transient inward current by isoproterenol in rabbit cardiac Purkinje fibers. Circ Res. 1995; 76: 664–674. 7895340 10.1161/01.res.76.4.664

[29] Morad M, Orkand RK. Excitation-concentration coupling in frog ventricle: evidence from voltage-clamp studies. J Physiol. 1971; 219: 167–189. 5316660 10.1113/jphysiol.1971.sp009656 PMC1331624

[30] Hinde AK, Perchenet L, Hobai IA, Levi AJ, Hancox JC. Inhibition of Na/Ca exchange by external Ni in guinea-pig ventricular myocytes at 37 degrees C, dialysed internally with cAMP-free and cAMP-containing solutions. Cell Calcium. 1999; 25: 321–331. 10456229 10.1054/ceca.1999.0035

[31] Bergson P, Lipkind G, Lee SP, Duban ME, Hanck DA. Verapamil block of T-type calcium channels. Mol Pharmacol. 2011; 79: 411–419. 10.1124/mol.110.069492 21149638 PMC3061365

