# Supplementary figures and images for: Excitation-Contraction Coupling in Zebrafish Ventricular Myocardium Is Regulated by Trans-Sarcolemmal Ca2+ Influx and Sarcoplasmic Reticulum Ca2+ Release
Source: PLoS One. 2015 May 4;10(5):e0125654. doi: 10.1371/journal.pone.0125654 (PMC4418605; doi:10.1371/journal.pone.0125654)

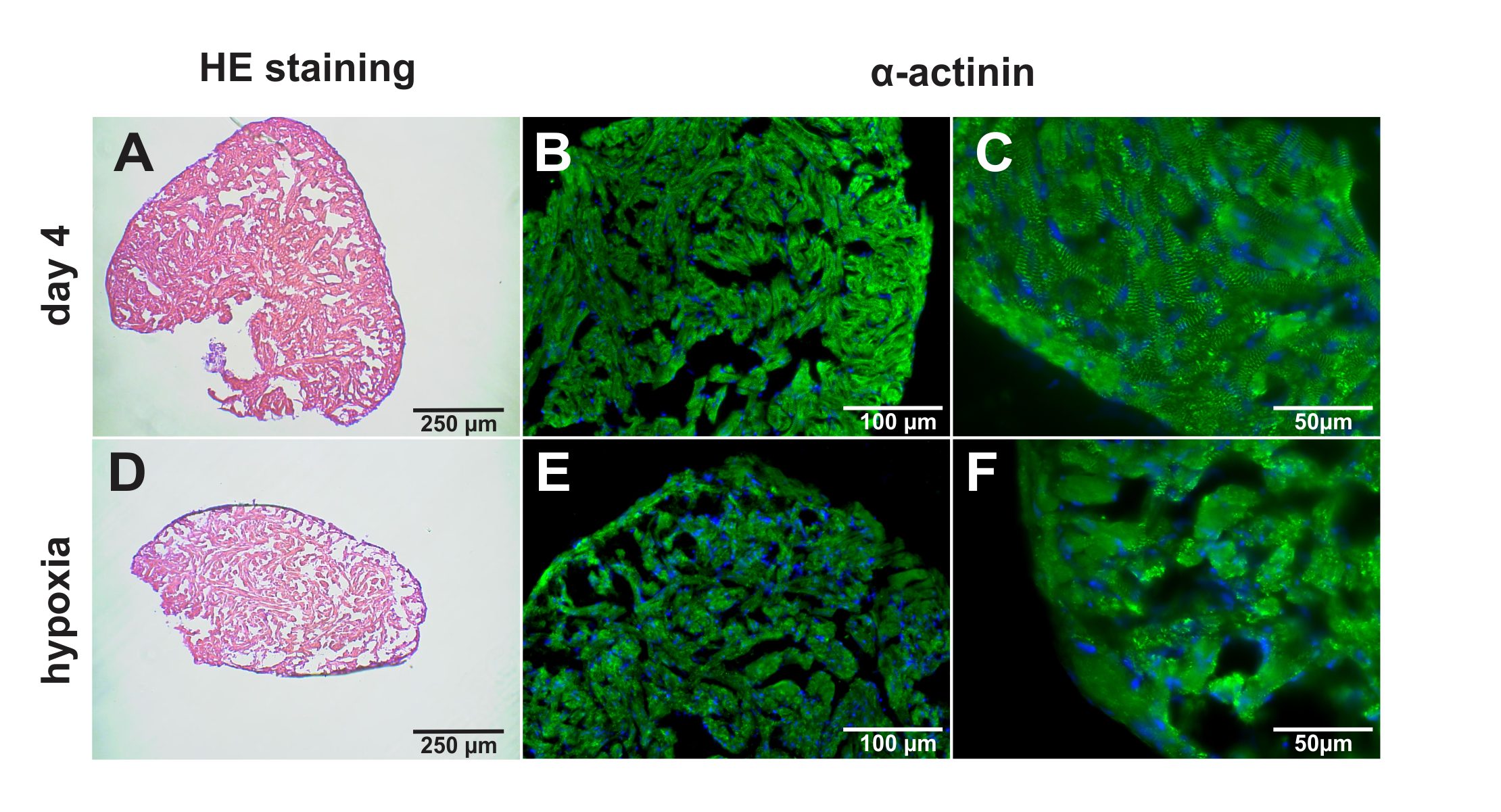

Supplement: S1 Fig — HE staining of cryosections (8 μm) obtained from a myocardial tissue slice at day 4 (A) and cultured under hypoxic conditions (1% O2 for 2 days) (D). Cyrosections stained against sarcomeric-α-actinin (green) at day 4 (B-C) or cultured under hypoxic conditions (E-F). Nuclei are counterstained with Hoechst (blue). (TIF) [file pone.0125654.s001.tif]

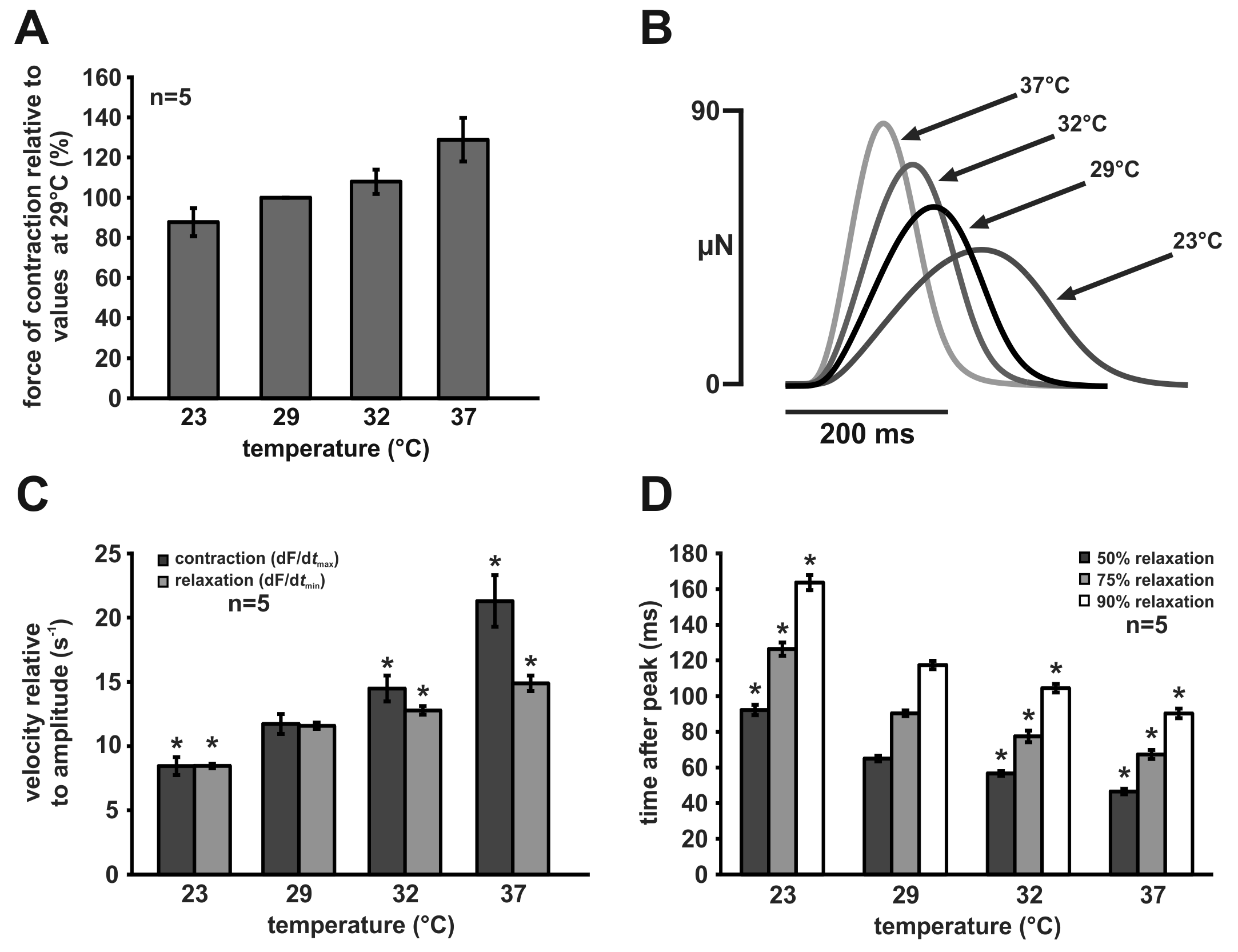

Supplement: S2 Fig — Box plots of the ratio of change in resting tension and change in amplitude after frequency switches. If frequency was reduced from 2 Hz to 1 Hz, a decrease in resting tension contributed to 16± 5% of amplitude increase, if increased from 1 Hz to 3 Hz, an increase in resting tension contributed to amplitude change by 6 ± 1%, and if decreased from 3 Hz to 2 Hz, resting tension contributed to 17 ± 4%. (TIF) [file pone.0125654.s002.tif]

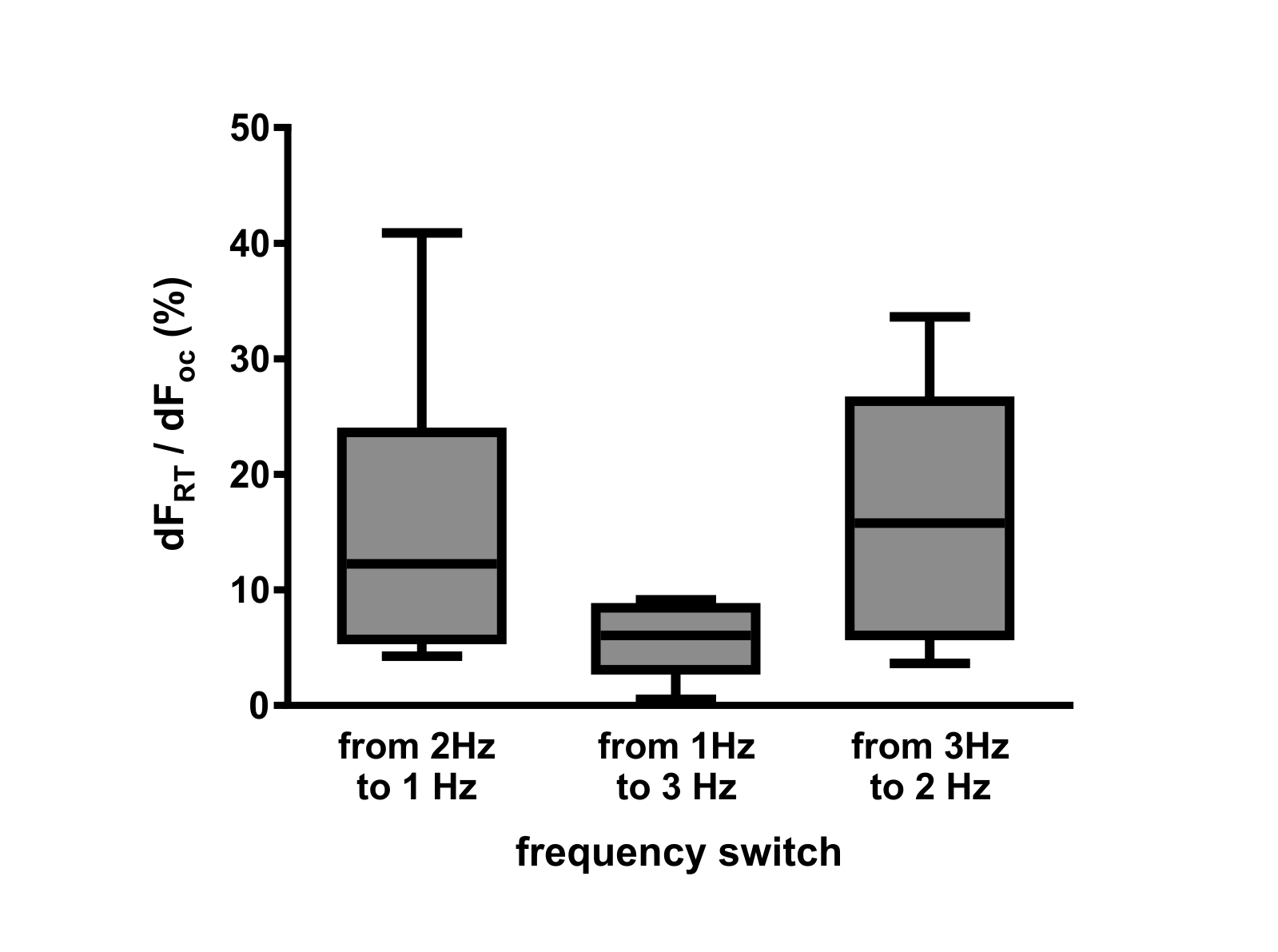

Supplement: S3 Fig — (A) Force of contraction increased with elevated temperature. Force of contraction was normalized to values at 29°C. Results are expressed as mean ± SEM (B) Original averaged twitches of a representative experiment showing the influence of investigated temperatures on twitch amplitude as well as contraction and relaxation kinetics. (C) Maximal contraction (dF/dt max) and relaxation (dF/dt min) velocities normalized to amplitude increased with temperature. (D) Relaxation of the myocardial slices was also accelerated at elevated temperatures. All data are expressed as mean ± SEM; asterisks indicate statistical significant differences (p < 0.05 vs. values at 29°C). (TIF) [file pone.0125654.s003.tif]
